# ZFP90 Serves as a Transcriptional Brake on NF-κB Signaling to Attenuate Diet-Induced MASLD Progression

**DOI:** 10.3390/nu18142332

**Published:** 2026-07-16

**Authors:** Seongjoon Park, Toshimitsu Komatsu, Kohei Misumi, Daisuke Okuzaki, Isao Shimokawa

**Affiliations:** 1 Department of Pathology, Graduate School of Biomedical Sciences, Nagasaki University School of Medicine, 1-12-4 Sakamoto, Nagasaki 852-8523, Japan; 2 Biomedical Research Support Center, Nagasaki University School of Medicine, Nagasaki 852-8523, Japan; 3 Laboratory for Human Immunology (Single Cell Genomics), WPI Immunology Frontier Research Center, The University of Osaka, Suita 565-0871, Japan; 4 Graduate School of Biomedical Sciences, Nagasaki University, Nagasaki 852-8523, Japan; 5 SAGL, Limited Liability Company, Fukuoka 810-0045, Japan

**Keywords:** MASLD, MASH, adipose tissue, liver, ZFP

## Abstract

**Background/Objectives:** Metabolic dysfunction-associated steatotic liver disease (MASLD) has become increasingly common, a trend driven by obesity, excess nutritional intake, and dysfunctional adipose tissue. While continuous dietary stress triggers adipose-tissue-derived lipotoxicity and disrupts hepatic metabolic homeostasis and provokes inflammation, the transcriptional scaffolds that mitigate this lipotoxicity remain incompletely understood. We investigated the role of zinc finger protein 90 (ZFP90) in defending against diet-induced metabolic stress and MASLD pathogenesis. **Methods:** Wild-type and ZFP90-knockout mice were subjected to a high-fat diet (HFD) to model nutrient-overload-induced MASLD. Hepatic phenotypes were characterized using metabolic profiling and RNA sequencing. Mechanistic dynamics were evaluated through protein interaction assays, and clinical relevance was validated using human MASLD liver biopsies. **Results:** ZFP90 deficiency significantly accelerated HFD-induced steatosis, systemic insulin resistance, and inflammatory infiltration. Crucially, ZFP90 depletion drove severe white adipose tissue (WAT) dysfunction, characterized by impaired lipogenic capacity, exacerbated lipolysis, and diminished local insulin signaling. This was accompanied by a pro-inflammatory secretory shift in WAT, evident from decreased Adipoq and increased Cd68/Ccl3 expression. In the liver, transcriptomic analysis revealed a profound induction of pathways related to fatty acid uptake and cytokine signaling. Mechanistically, ZFP90 forms a repressive complex with TRIM28, acting as a crucial molecular brake on NF-kB signaling. Loss of ZFP90 unleashes p65-mediated hyper-inflammation. Clinically, hepatic ZFP90 expression is significantly upregulated in patients with MASLD. **Conclusions:** ZFP90 is a novel regulator of immunometabolic homeostasis under dietary stress. By forming of complex with Trim28 to inhibit the nuclear translocation of NF-κB, ZFP90 suppresses pro-inflammatory responses and protects the liver from obesity-associated systemic lipotoxicity. These findings provide critical insights into the adipo-hepatic axis and highlight ZFP90 as a promising therapeutic target to mitigate the progression to metabolic dysfunction-associated steatohepatitis (MASH).

## 1. Introduction

Chronic liver disease prevalence has reached a critical global threshold, with metabolic dysfunction-associated steatotic liver disease (MASLD), previously referred to as non-alcoholic fatty liver disease (NAFLD), impacting more than 30% of the worldwide population [1]. Its prevalence is increasing in parallel with obesity and type 2 diabetes, reflecting the broader expansion of metabolic syndrome driven by chronic nutrient overload [2,3]. Defined by lipid accumulation in over 5% of hepatocytes, MASLD is primarily associated with metabolic disturbances like insulin resistance and obesity, provided that secondary liver pathologies or alcohol overconsumption are excluded.

The pathological spectrum of MASLD is extensive, progressing from simple steatosis to metabolic dysfunction-associated steatohepatitis (MASH), which is defined by varying degrees of inflammation and fibrosis that can culminate in cirrhosis and hepatocellular carcinoma (HCC) [4,5,6]. Beyond hepatic outcomes, MASLD significantly elevates the risk of extrahepatic complications, including cardiovascular and chronic kidney disease. Despite this substantial clinical and socioeconomic burden, pharmacological interventions remain limited, emphasizing the urgent need to elucidate the molecular drivers governing its pathogenesis [7]. Current paradigms suggest that MASLD progression is orchestrated by a “multiple-hit” process. Central to this process is the impact of continuous dietary stress, which precipitates adipose tissue dysfunction, impairs intestinal barrier integrity, and ultimately leads to the chronic activation of hepatic immune cell populations [8].

In particular, the adipo-hepatic axis plays a pivotal role in determining the liver’s response to nutritional stress. As the largest immunological organ in the body, the liver hosts a diverse repertoire of innate and adaptive immune cells, including Kupffer cells, macrophages, and lymphocytes [9]. Substantial alterations in these immune populations have been observed in both murine and human MASLD, contributing to a dysregulated inflammatory milieu that exacerbates hepatocellular injury [10,11,12]. Recent evidence highlights that obesity-induced adipose tissue dysfunction—characterized by lipolysis and pro-inflammatory adipokine secretion—creates a state of systemic lipotoxicity. This systemic stress, combined with intrahepatic immune-parenchymal crosstalk, coordinates the delicate balance between lipid metabolism and inflammatory signaling [13,14,15,16]. While perturbations in these cellular interactions are recognized as the primary drivers of MASLD progression [16], the specific molecular mediators that integrate systemic metabolic cues with local immune regulation remain largely elusive.

Zinc finger proteins (ZFPs), the most expansive family of transcriptional regulators, are indispensable for diverse biological processes, ranging from cellular differentiation to immune modulation [17,18]. Although select ZFP family members have been implicated in metabolic homeostasis [19,20,21], the functional role of zinc finger protein 90 (ZFP90) remains poorly understood. While ZFP90 has been previously associated with hematopoietic cell proliferation and cardiac development [22,23], its potential involvement in systemic adipo-hepatic and immune–metabolic regulation has yet to be explored.

In the present study, we identified ZFP90 as a critical regulator of systemic and hepatic immune-metabolic homeostasis under dietary stress. By employing a ZFP90-deficient murine model subjected to a diet high in fat, integrated transcriptomic profiling, and mechanistic investigations of the ZFP90-TRIM28 interaction, we demonstrate that ZFP90 deficiency drives profound white adipose tissue (WAT) dysfunction, exacerbates hepatic lipotoxicity, alters intrahepatic T-cell dynamics, and promotes NF-κB–dependent hyper-inflammation. Our results establish ZFP90 as an unconventional regulator of MASLD progression, identifying this protein as a promising candidate for therapeutic interventions aimed at stalling MASH development under conditions of chronic nutritional stress.

## 2. Materials and Methods

### 2.1. Animal Models and Diets

All animal procedures and experimental protocols were strictly performed in accordance with the guidelines approved by the Animal Care and Use Committee of Nagasaki University (Protocol No. 1909171569, approval date: 1 July 2024; and 2404301945, approval date: 30 April 2024). Zfp90-knockout (Zfp90−/−) mice on a C57BL/6 background were generated by deleting exon 3 of the mouse *Zfp90* transcript at the Research Center for Biomedical Models and Animal Welfare, Nagasaki University. Genotypes were confirmed by PCR analysis of ear punch biopsies. Wild-type (WT) littermates were used as controls throughout the study. Animals were maintained in a specific pathogen-free (SPF) facility under a controlled environment (temperature: 21–24 °C; humidity: 40–60%; 12 h light/dark cycle). Health monitoring was routinely performed according to institutional SPF guidelines, and no adverse events were reported during the study period. Mice were group-housed (3 mice per cage) in standard microisolator cages with wood-chip bedding. After a 2-week acclimatization period, 12-week-old male mice were randomly assigned to either a standard chow diet (CRF-1, Oriental Yeast Co., Ltd., Tokyo, Japan) or a high-fat diet (HFD-60; 60% kcal from fat, Oriental Yeast Co., Tokyo, Japan) for 20 weeks to model nutrient overload. To ensure baseline comparability, mice were stratified by initial body weight, and group assignment was determined using a randomization sequence. To minimize potential confounders from microenvironmental factors, cage positions on the housing racks were systematically rotated weekly. Due to the obvious physical differences between the chow and HFD pellets, allocation concealment and blinding of the personnel administering the diets were not feasible. The individual mouse was considered the experimental unit for body weight and molecular analyses, whereas the cage served as the experimental unit for food intake measurements. To evaluate the effects of genotype, feeding, and aging, food intake was analyzed using three-way analysis of variance (ANOVA) across 12, 15, 20, 24, and 28 weeks of age. Animal welfare and body weight were monitored regularly. Humane endpoints were pre-defined as (>20% body weight loss, severe lethargy, or inability to access food/water), though no animals reached these criteria. For terminal tissue collection, mice in the *ad libitum* fed state were deeply anesthetized with isoflurane (FUJIFILM Wako Pure Chemical Corp., Osaka, Japan). Following blood collection via cardiac puncture, mice were euthanized by exsanguination, and tissues were immediately harvested and snap-frozen in liquid nitrogen. Full procedural details regarding in vivo micro-CT, blood sampling, glucose tolerance tests (GTT), LPS injection, and non-parenchymal cell (NPC) isolation are described in their respective sections below.

### 2.2. In Vivo Micro-Computed Tomography (Micro-CT) Analysis

In vivo body composition, specifically the quantification of total, visceral, and subcutaneous adipose tissue, was evaluated at 6 months of age using a 3D micro-computed tomography (micro-CT) system (Rigaku Co., Tokyo, Japan). Briefly, mice were anesthetized via an intraperitoneal injection of a triple-anesthetic mixture (0.3 mg/kg body weight medetomidine hydrochloride, 4.0 mg/kg midazolam, and 5.0 mg/kg butorphanol tartrate) at a dose of 10 µL/g body weight, and placed in a prone position on the scanning bed. Scans were acquired covering the abdominal region (from the diaphragm to the pelvic floor) using the following parameters: a tube voltage of 60 kV, a tube current of 60 µA. The acquired tomographic images were reconstructed and analyzed using Rigaku 3D-CT imaging software (Analyze v11.0). Adipose tissue was segmented based on established radiodensity thresholds (Hounsfield Units). Visceral and subcutaneous fat compartments were anatomically separated by manually delineating the abdominal muscle wall boundaries across the serial axial slices. The calculated fat volumes were converted to mass and expressed as a percentage of total body weight.

### 2.3. Metabolic and Biochemical Phenotyping

Whole-body energy consumption (VO2), carbon dioxide production (VCO2), and respiratory quotient (RQ) were monitored using an indirect calorimetry system (MK-5000RQ, Muromachi Kikai Co., Tokyo, Japan). Glucose homeostasis was evaluated via glucose tolerance tests (GTT); following a 4 h fast, mice received an intraperitoneal injection of glucose (1 g/kg body weight). Blood samples were collected from the tail vein, and glucose levels were measured at 0, 15, 30, 60, and 120 min post-injection using an Accu-Chek Aviva Nano Meter (Roche Diagnostics, Tokyo, Japan). Serum concentrations of insulin, alanine aminotransferase (ALT), and aspartate aminotransferase (AST) were quantified using validated ELISA systems (Morinaga Institute of Biological Science, Yokohama, Japan; FUJIFILM Wako Pure Chemical Corp., Osaka, Japan) according to the manufacturers’ protocols.

### 2.4. Liver Non-Parenchymal Cell (NPC) Isolation

To evaluate the *in vivo* inflammatory response, mice received an intraperitoneal injection of lipopolysaccharide (LPS, 100 µg/kg body weight) or an equivalent volume of saline. Two hours post-injection, mice were euthanized for hepatic non-parenchymal cell (NPC) isolation. Briefly, mice were perfused through the left ventricle with 20 mL of phosphate-buffered saline (PBS) using a 25-gauge needle to clear blood from the tissue. The liver was then excised, washed with PBS, and mechanically minced into 1–2 mm pieces using surgical scissors. The minced tissue (approximately 0.5–1.5 g) was transferred into a 50 mL conical tube containing digestion buffer supplemented with Collagenase Type IV and incubated at 37 °C for 1 h with constant shaking at 220 rpm. During the incubation, the cell suspension was vortexed every 10 min to facilitate enzymatic dissociation. Following digestion, EDTA (50 mM) was added to a final concentration of 10 mM, and the mixture was further incubated at 37 °C for 5 min. The digested cell suspension was then passed through a pre-wetted 70 µm nylon cell strainer, and 10–15 mL of culture medium supplemented with 2% fetal bovine serum (FBS) was added to terminate the enzymatic reaction. To separate the NPCs from hepatocytes, the suspension was centrifuged at 50× *g* for 5 min at 4 °C with the centrifuge brake turned off. The resulting pellet, which primarily containing hepatocytes, was discarded. The supernatant, comprising the enriched NPC fraction, was collected and subsequently centrifuged at 500× *g* for 7 min at 4 °C (without brake). The resulting NPC pellet was resuspended in 500 µL of red blood cell (RBC) lysis buffer and incubated for 5 min at room temperature. Finally, the cells were pelleted by a final centrifugation step at 500× *g* for 7 min at 4 °C (without brake), yielding the purified NPC fraction for subsequent analyzes.

### 2.5. Mass Spectrometry Analysis

Protein samples were fractionated on a 4–20% gradient SDS-PAGE (Cosmo Bio, Tokyo, Japan) and stained with GelCode Blue Reagent (Thermo Fisher Scientific, Waltham, MA, USA). Protein bands ranging from 13 to 120 kDa were excised and subjected to in-gel digestion according to the manufacturer’s instructions (Bruker Daltonics, Bremen, Germany). Briefly, the gel pieces were destained with 25 mM ammonium bicarbonate in 50% acetonitrile (ACN), reduced with 10 mM dithiothreitol at 56 °C for 45 min, and alkylated with 55 mM iodoacetamide for 30 min at room temperature. After dehydration with ACN, the gel was incubated with 20 ng/µL Trypsin Gold (Promega, Madison, WI, USA) at 37 °C overnight. Digested peptides were extracted using 50% ACN/5% trifluoroacetic acid (TFA), followed by sonication and vortexing. The extract was concentrated by evaporation and reconstituted in 2% ACN/0.1% TFA. Protein analysis was performed via an LTQ-XL LC-ESI-MS/MS platform configured to our specifications (Thermo Fisher Scientific). Raw MS/MS data were processed and searched against the UniProt *Mus musculus* database by the Proteome Discoverer software suite, version 3.3 (Thermo Fisher Scientific) to identify high-confidence protein–protein interactions.

### 2.6. RNA Sequencing and Transcriptomic Analysis

Library preparation was performed using a TruSeq Stranded mRNA Sample Prep Kit (Illumina, San Diego, CA, USA) according to the manufacturer’s instructions. Sequencing was performed on an Illumina NovaSeq 6000 platform in 151-base single-end mode. The sequenced reads were trimmed using Trimmomatic v.0.39, with subsequent alignment to the GRCm38.p4 mouse genome assembly performed via HISAT2 v.2.1.0. Fragments per kilobase of exon per million mapped fragments (FPKMs) were calculated using Cufflinks version 2.0.6. Differentially expressed genes (DEGs) were determined based on a fold-change threshold of >2.0 or <0.5, with a significance level of *p* < 0.05. Raw data were deposited in the NCBI Gene Expression Omnibus (GEO) under accession number GSE324098.

### 2.7. Quantitative Real-Time PCR

Liver and adipose tissue samples were processed for total RNA isolation following the standard protocol provided with the RNeasy Tissue Kit (Qiagen, Hilden, Germany). cDNA was synthesized from the isolated RNA using the ReverTra Ace qPCR RT Kit (Toyobo, Osaka, Japan). mRNA abundance levels were assessed through quantitative real-time PCR (qPCR) using the THUNDERBIRD™ SYBR and probe qPCR Mix (Toyobo, Japan) on a real-time PCR detection system. The reaction conditions followed the standardized thermal cycling protocol provided by the manufacturer. The expression levels of mouse *Zfp90* and human *ZFP90* were analyzed using TaqMan probes (Mm00496071_m1 and Hs01001573_g1, respectively). Relative mRNA expression levels were calculated and normalized to the endogenous controls, 18S ribosomal RNA (18S), ribosomal protein lateral stalk subunit P0 (*Rplp0*), and glyceraldehyde-3-phosphate dehydrogenase (Gapdh). All primer sequences used in this study are listed in Tables S1 and S2.

### 2.8. Western Blotting

To assess the nuclear translocation of NF-κB, nuclear and cytoplasmic protein fractions were isolated from frozen liver tissues using the NE-PER™ Nuclear and Cytoplasmic Extraction Reagents (Thermo Fisher Scientific, MA, USA) according to the manufacturer’s instructions. Briefly, liver tissues were mechanically homogenized in Cytoplasmic Extraction Reagent I (CER I) supplemented with protease and phosphatase inhibitors. Following a brief incubation and the addition of CER II, the homogenates were centrifuged to collect the supernatant containing the cytoplasmic fraction. The remaining insoluble pellet was then resuspended in Nuclear Extraction Reagent (NER), vortexed iteratively, and centrifuged to collect the highly enriched nuclear fraction. Proteins were separated by sodium dodecyl sulfate-polyacrylamide gel electrophoresis (SDS-PAGE) and transferred onto nitrocellulose membranes. After blocking for 60 min in TBS-T buffer supplemented with either 5% non-fat milk or 3% BSA, membranes were incubated overnight at 4 °C with specific primary antibodies. The antibodies used included anti-p65, anti-IkBα, anti-TRIM28, and anti-p-AKT (Ser473) (Cell Signaling Technology, Danvers, MA, USA), anti-Flag (Sigma-Aldrich, St. Louis, MO, USA), as well as anti-Lamin B1 and anti-β-actin (Abcam, Cambridge, UK). Following incubation with species-specific HRP-linked secondary antibodies (Amersham Pharmacia Biotech, Amersham, UK), Protein detection was achieved using standard enhanced chemiluminescence (ECL) detection kits (Thermo Fisher Scientific, MA, USA). Signals were quantified via the FluorChem system (Alpha Innotech Corp., San Leandro, CA, USA) using AlphaEase FC software (v4.0).

### 2.9. Histological Analyzes

Histological evaluation involved fixing liver and fat tissues in 4% PFA, paraffin embedding, and subsequent hematoxylin and eosin (H&E) staining. Images were acquired using a BZ-X700 microscope (Keyence, Osaka, Japan). Quantitative analysis of adipocyte size and brown adipocyte count was performed using the BZ-X700 application software (BZ-X Analyzer v1.4.1.1) and ImageJ (1.54t) (*n* = 4 per group). To assess collagen deposition and fibrosis, liver tissue sections were subjected to Masson’s Trichrome staining. Briefly, deparaffinized sections were treated with Mordant I for 30 min, followed by staining with Carazzi’s hematoxylin for 45 min. Subsequent staining steps involved Orange G, Masson B, and Aniline blue, with 1% acetic acid rinses applied between each reagent step. Images of the stained sections were acquired using a BZ-X700 microscope (Keyence, Osaka, Japan), and quantitative analysis was conducted using the BZ-X700 application software and ImageJ (*n* = 4 per group).

### 2.10. Cell Culture

COS-7 cell maintenance was performed in DMEM (Dulbecco’s Modified Eagle’s Medium). RPMI medium supplemented with 10% FBS, 100 mg/mL streptomycin, and 100 U/mL penicillin served as the culture environment for MT-2 and SKW-3 cell lines. Cells were incubated at 37 °C in humidified 5% CO_2_ and subcultured upon reaching confluence.

### 2.11. Clinical Study

Formalin-fixed paraffin-embedded (FFPE) human liver sections were obtained from patients with MASLD and healthy liver transplant donors at Nagasaki University Hospital (2010–2024). Total RNA was isolated using the RNeasy FFPE Kit (Qiagen, Germany) and cDNA was synthesized as described above. Clinical and biochemical characteristics of the study population were obtained from the Nagasaki University Hospital information system. All procedures were performed in accordance with protocols approved by the Clinical Research Ethics Committee of Nagasaki University Hospital on 31 July 2005 (Permit No. 25080703, approval date: 31 July 2025).

### 2.12. Statistical Analysis

To prevent observer bias, all collected samples were numerically coded. The investigators involved in histological scoring, image quantification, and downstream biochemical analyses were strictly blinded to the genotype and dietary treatment groups of the animals until the completion of the data analysis. Data are expressed as means ± SEM. Statistical significance was assessed by unpaired two-tailed Student’s *t*-test or two-/three-way ANOVA, followed by Bonferroni’s multiple comparisons test. Analysis was performed using GraphPad Prism 5, with *p* < 0.05 considered statistically significant.

## 3. Results

### 3.1. ZFP90 Deficiency Promotes Visceral Adiposity and Shifts Metabolic Substrate Utilization

**Figure 1:**
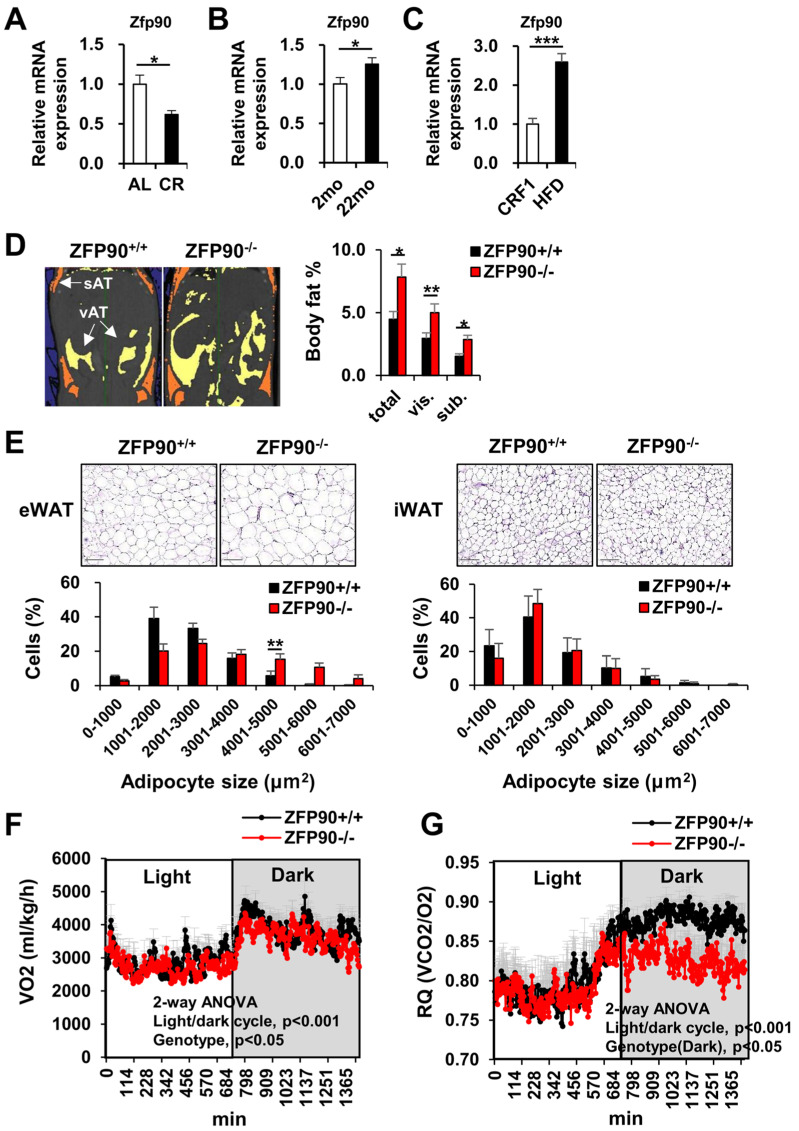
ZFP90 deficiency increases adiposity in mice. (**A**–**C**) mRNA expression of Zfp90 in (**A**) caloric restricted (CR), (**B**) aged, and (**C**) HFD-fed mice (*n* = 5–6 per group). (**D**) Representative 3D-Micro CT images of subcutaneous and visceral adipose tissue (left) and quantification of total, visceral (vis), and subcutaneous (sub) adipose tissue percentages (right) (*n* = 7 per group). (**E**) Representative hematoxylin and eosin (H&E) staining of epididymal (eWAT) and inguinal (iWAT) white adipose tissue (upper). Scale bar, 100 μm. Mean adipocyte sizes from eWAT and iWAT are shown in the bottom panel (*n* = 4 per group). (**F**) Oxygen consumption (VO_2_) and (**G**) respiratory quotient (RQ) in WT and KO mice fed a HFD (*n* = 4 per group). Data are expressed as means ± SEM. Statistical significance was assessed by unpaired two-tailed Student’s *t*-test or two -way ANOVA, followed by Bonferroni’s multiple comparisons test; * *p* < 0.05, ** *p* < 0.01, and *** *p* < 0.001. Abbreviations: AL, ad libitum; mo, month; sAT, subcutaneous adipose tissue; vAT, visceral adipose tissue.

**Figure 2:**
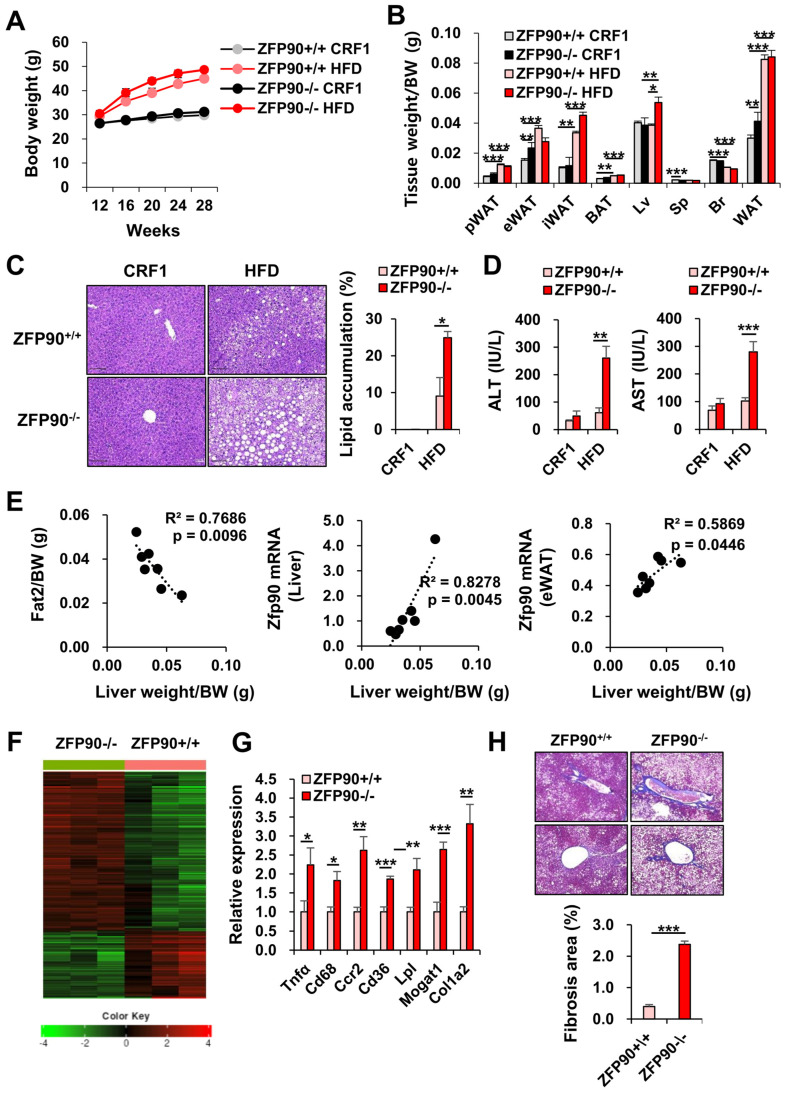
ZFP90 deletion exacerbates HFD-induced MASLD. (**A**) Body weight and (**B**) tissue weight changes during 16 weeks of CRF1 (control) or HFD feeding in WT and KO mice (*n* = 7 per group). (**C**) Representative H&E staining of liver sections (left; scale bar, 100 μm) and quantification of lipid accumulation in WT and KO mice (right) (*n* = 4 per group). (**D**) Serum ALT and AST levels (*n* = 5–7 per group). (**E**) Correlation analysis: epididymal fat mass/BW ratio vs. liver weight/BW ratio (left); *Zfp90* mRNA levels in the liver or eWAT vs. liver weight/BW ratio in HFD-fed mice (right) (*n* = 6–7 per group). (**F**) Heatmaps showing differentially expressed genes (DEGs) from the transcriptomic analysis of livers from HFD-fed mice (*n* = 3 per group). (**G**) mRNA expression of genes related to fatty acid uptake (*Cd36*, *Lpl*, *Mogat1*), inflammation (*Tnfa*, *Cd68*, *Ccr2*), and fibrosis (*Col1a2*) in the liver (*n* = 6–7 per group). (**H**) Representative Masson’s trichrome staining of liver sections (upper; scale bar, 100 μm) and quantification of the fibrosis area (bottom) (*n* = 4 per group). Statistical significance was determined by three-way ANOVA with Bonferroni’s multiple-comparisons test or Student’s two-tailed *t*-test; * *p* < 0.05, ** *p* < 0.01, and *** *p* < 0.001. Data are expressed as means ± SEM. Abbreviations: BAT, brown adipose tissue; Br, brain; eWAT, epididymal white adipose tissue; I, inguinal; pWAT, perirenal, inguinal; Sp, spleen.

We previously demonstrated that neuropeptide Y (NPY)-mediated lipid metabolic balance is essential for calorie restriction-induced lifespan extension, and that zinc finger protein 90 (ZFP90) serves as a key mediator in this NPY-related lipid metabolism [24]. We found that *Zfp90* mRNA expression in white adipose tissue (WAT) was downregulated under a negative energy balance (30% CR; Figure 1A) but significantly upregulated under a positive energy balance (chronic nutrient overload via high-fat diet, HFD) or aging (Figure 1B,C). To investigate its functional role, 12-week-old ZFP90 wild-type (WT) and knockout (KO) mice were fed either a standard chow diet (CRF1) or an HFD for 20 weeks. Three-way ANOVA analysis revealed distinct patterns in both food intake and weight gain across groups. Regarding food intake, while diet (Feeding, *p* < 0.0001) and age (Weeks, *p* = 0.0006) were significant determinants, genotype did not show a main effect; however, a significant Genotype × Feeding interaction (*p* = 0.0017) indicates that ZFP90 deficiency modulates the dietary effect on food intake (Figure 2A and Figure S1). This suggests that ZFP90 is involved in the adaptive response to dietary challenges, with its absence altering the metabolic regulation of energy intake. Parallel to these findings, longitudinal monitoring of body weight showed that genotype (*p* = 0.0006), feeding (*p* < 0.0001), and age (*p* < 0.0001) all exerted significant effects on weight gain. Notably, the significant Genotype × Feeding interaction (*p* = 0.0227) and the Feeding × Weeks interaction (*p* < 0.0001) underscore that ZFP90 deficiency significantly modulates the weight gain trajectory, particularly under high-fat dietary stress (Figure 2A). Despite these metabolic differences, body fat mass was significantly higher in KO mice fed the CRF1 diet (Figure 1D). Histological analysis revealed larger adipocytes in the epididymal WAT (eWAT) of KO mice compared to WT mice fed the CRF1 diet (Figure 1E). These results indicate that ZFP90 deficiency promotes visceral adiposity.”

### 3.2. Loss of ZFP90 Exacerbates HFD-Induced MASLD and Hepatic Transcriptomic Remodeling

On the HFD, both WT and KO mice gained significantly more weight than those on the chow diet, with no significant inter-genotype differences in weight gain (Figure 2A), total WAT accumulation (Figure 2B), and adipocyte size (Figure S2A,B). However, KO mice exhibited exacerbated liver injury under HFD, as evidenced by significantly elevated serum alanine aminotransferase (ALT) and aspartate aminotransferase (AST) levels (Figure 2D). Regression analysis revealed a strong positive correlation between visceral fat weight and the liver weight-to-body weight (BW) ratio, underscoring that excessive adiposity and systemic lipid overflow directly drive hepatic steatosis (Figure 2C,E). Notably, *Zfp90* mRNA expression in eWAT was positively correlated with the liver-to-BW ratio, suggesting its role as a reactive regulator against systemic lipid stress (Figure 2E).

Transcriptomic profiling (RNA-seq) identified 1038 upregulated and 263 downregulated genes (fold-change > 2) in ZFP90-KO livers compared to WT livers (Figure S3A). Pathway enrichment analysis revealed that ZFP90 deficiency upregulated programs involved in immunity, inflammatory responses, and lipid metabolism—specifically cytokine-cytokine receptor interactions, NF-kB, and chemokine signaling (Figure S3B). To explore the mechanisms underlying ZFP90 deficiency-induced hepatic steatosis, we assessed specific hepatic gene expression based on the RNA-seq data. ZFP90 deficiency increased the mRNA levels of genes related to fatty acid uptake (*Cd36*, *Lpl*, *Mogat1*), inflammation (*Tnfa*, *Cd68*, *Ccr2*), and fibrosis (*Col1a2*) (Figure 2F,G). These findings indicate that the loss of ZFP90 accelerates diet-induced MASLD progression through the integrated dysregulation of inflammatory and metabolic pathways. Consistent with these molecular findings, histological examination via Masson’s trichrome staining further corroborated the structural divergence between the genotypes under HFD feeding (Figure 2H). While ZFP90-WT mice displayed collagen deposition primarily restricted to the portal areas, ZFP90-KO mice exhibited a more advanced pathological state, characterized by exacerbated portal fibrosis that extended into the peri-portal regions. Collectively, these histological observations provide visual confirmation that the loss of ZFP90 facilitates the structural progression of fibrosis, reinforcing its role in accelerating diet-induced MASLD. These findings suggest that ZFP90 may function as a crucial homeostatic regulator that limits excessive extracellular matrix remodeling under chronic metabolic stress. The transition from restricted portal deposition in wild-type mice to more extensive peri-portal fibrosis in ZFP90-deficient mice highlights the exacerbated fibrogenic signaling that occurs in the absence of ZFP90.

### 3.3. ZFP90-Deficiency Drives Adipose Tissue Dysfunction and Systemic Insulin Resistance

**Figure 3:**
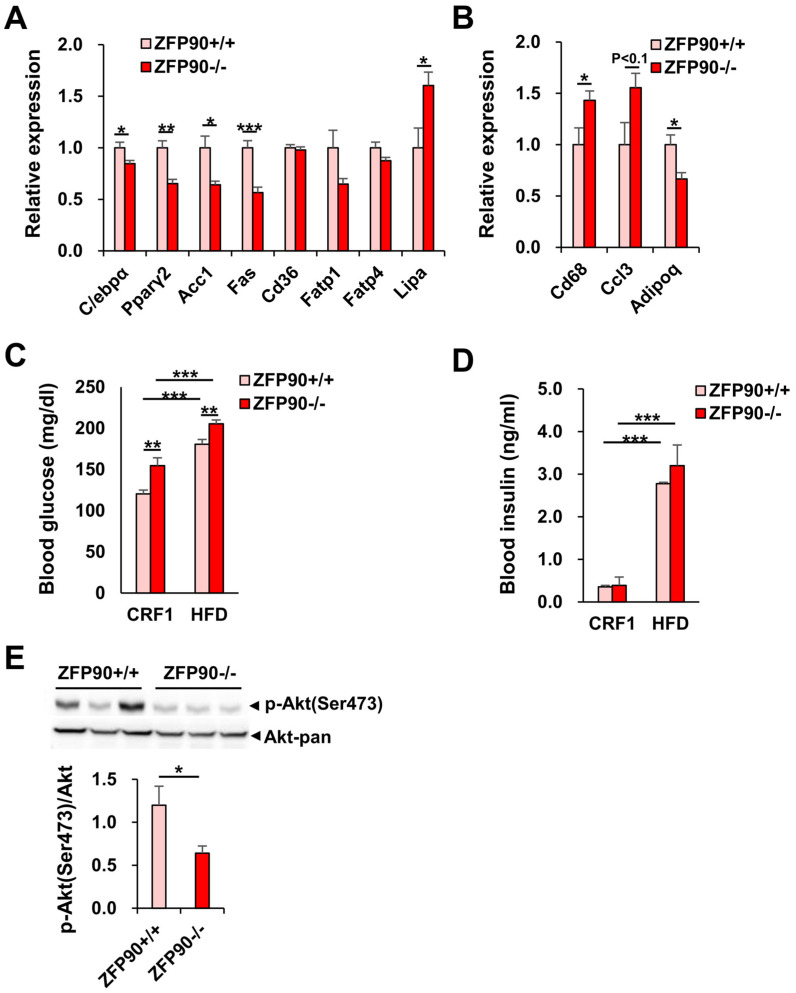
ZFP90-deficiency induces adipose tissue dysfunction and insulin resistance. (**A**,**B**) mRNA expression of genes related to (**A**) lipogenesis (*Cebpa*, *Pparg2*, *Acc1*, *Fasn*), lipolysis (*Lipa*), (**B**) pro-inflammatory markers (*Cd68*, *Ccl3*), and *Adipoq* in eWAT (*n* = 6–7 per group). (**C**) Fasting blood glucose, and (**D**) insulin levels in WT and KO mice (*n* = 6–7 per group). (**E**) Protein levels of *p*-Akt (ser473) and Akt (upper) measured by Western blot in eWAT collected from mice in the fed state, with quantification (bottom) (*n* = 4 per group). Data are expressed as means ± SEM. Statistical significance was determined by Student’s two-tailed *t*-test; * *p* < 0.05, ** *p* < 0.01, and *** *p* < 0.001.

Adipose tissue dysfunction and systemic inflammation are known primary drivers of hepatic steatosis [25]. In HFD-fed mice, ZFP90 deficiency significantly decreased the expression of key lipogenic genes (*Cebpa*, *Pparg2*, *Acc1*, and *Fasn*) while increasing the expression of the lipolytic gene *Lipa* in white adipose tissue (WAT) (Figure 3A). This imbalance suggests an impaired lipid storage capacity in WAT, which potentially exacerbates ectopic lipid deposition in the liver. Furthermore, ZFP90-KO mice exhibited a pronounced pro-inflammatory shift in WAT, indicated by the upregulation of macrophage and inflammatory markers (*Cd68* and *Ccl3*) and the concurrent downregulation of the anti-inflammatory adipokine *Adipoq* (Figure 3B). While both genotypes developed HFD-induced hyperinsulinemia, ZFP90-KO mice exhibited markedly higher fasting blood glucose levels and diminished Akt phosphorylation (*p*-Akt(Ser473)) in WAT (Figure 3C–E). To further evaluate the impact of ZFP90 deficiency on systemic glucose handling, we performed glucose tolerance tests (GTT). Three-way ANOVA analysis revealed that both dietary intervention (Feeding, *p* = 0.0019) and time post-glucose injection (Time, *p* < 0.0001) significantly influenced blood glucose levels. While dietary stress profoundly altered glucose tolerance, no statistically significant interaction was observed between genotype and diet or time, suggesting that the systemic glucose intolerance in this model is primarily driven by nutrient overload rather than genotype alone (Figure S4). Collectively, these results demonstrate that ZFP90 deficiency exacerbates MASLD pathogenesis, at least in part, by driving adipose tissue dysfunction and impairing systemic insulin sensitivity. It is important to note that the hepatic lipid accumulation observed in our 20-week HFD model represents simple steatosis rather than metabolic dysfunction-associated steatohepatitis (MASH). Despite the significant increase in hepatic lipid content and *Cd36* expression in HFD-fed mice compared to the control group, we observed no statistically significant upregulation in pro-inflammatory markers such as *Tnfa* and *F4/80* within the liver tissue (Figure S5). Furthermore, serum ALT and AST levels remained within the normal range (Figure 2D), indicating an absence of significant hepatocellular injury. These findings suggest that in our experimental model, the exacerbated hepatic lipid deposition driven by HFD occurs primarily through an adipose-liver axis, as evidenced by compromised lipid storage in WAT and subsequent ectopic lipid overflow, rather than through a direct pro-inflammatory progression of the liver toward MASH.

### 3.4. ZFP90 Interacts with TRIM28 to Suppress NF-κB Dependent Hepatic Inflammation

**Figure 4:**
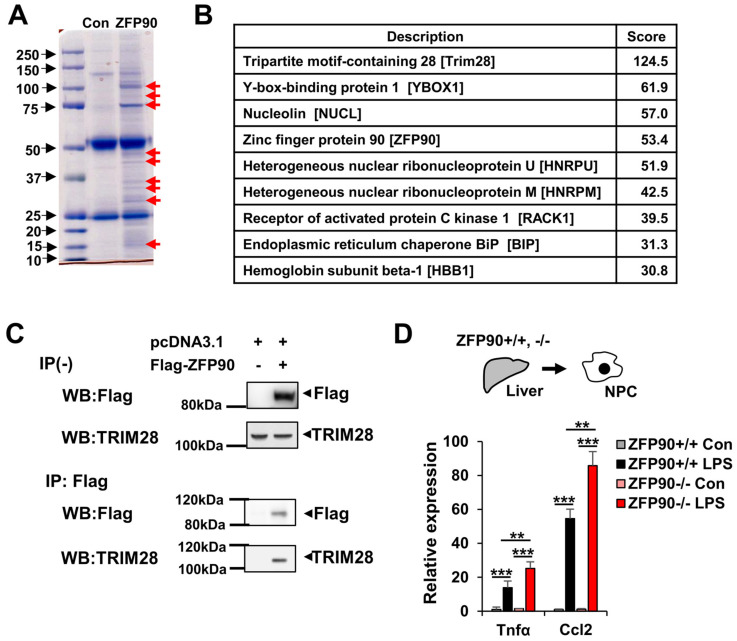
ZFP90 interacts with TRIM28 to modulate NF-κB activation. (**A**–**C**) Co-immunoprecipitation (Co-IP) analysis in COS-7 cells. Cells were transfected with FLAG-tagged ZFP90 and lysed 48 h post-transfection. Cell lysates were subjected to immunoprecipitation with an anti-Flag antibody, followed by (**A**) Coomassie Blue-stained SDS-PAGE to visualize ZFP90-binding proteins. (**B**) Arrows and letters indicate bands excised for LC-MS/MS identification. Molecular weight markers (kDa) are shown on the left. (**C**) Representative immunoblots of FLAG-immunoprecipitated proteins and input lysates using anti-FLAG and anti-Trim28 antibodies. (**D**) mRNA expression of *Tnfα* and *Ccl2* in isolated non-parenchymal cells from WT and KO mice treated with LPS (0.1 mg/kg) or saline for 2 h (*n* = 3–9 per group). Data are expressed as means ± SEM. Statistical significance was determined by Student’s two-tailed *t*-test; ** *p* < 0.01 and *** *p* < 0.001.

To identify ZFP90-binding partners and elucidate its molecular mechanisms, we performed immunoprecipitation and LC-MS/MS analysis in COS-7 cells expressing FLAG-tagged ZFP90 (Figure 4A). Ten protein bands containing putative ZFP90-binding candidates were used to identify nine potential interacting proteins (Figure 4A,B). Among them, tripartite motif-containing 28 (TRIM28) is of particular interest, as previous reports have suggested that ZFP90 forms a complex with TRIM28, a known regulator of NF-κB signaling [26,27]. To validate this interaction, Western blot analysis was performed, confirming the physical association between ZFP90 and TRIM28 (Figure 4C). To test the functional impact of this interaction within the hepatic microenvironment, we stimulated liver non-parenchymal cells from LPS-injected mice. LPS-induced *Tnfa* and *Ccl2* expression was significantly higher in ZFP90-KO cells, demonstrating that the ZFP90-TRIM28 complex serves as a critical suppressor of excessive NF-κB activation (Figure 4D).

### 3.5. ZFP90 Deficiency Alters T-Cell Dynamics

**Figure 5:**
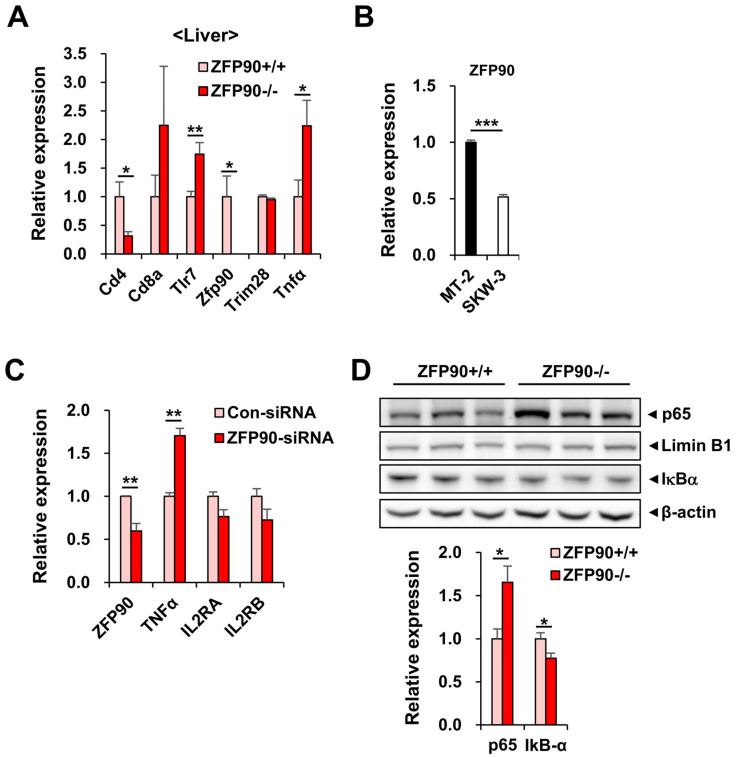
ZFP90 deficiency alters intrahepatic T-cell dynamics. (**A**) mRNA expression of genes related to T-cell dynamics (*Cd4, Cd8a, Tlr7*), *Zfp90*, and *Trim28* in eWAT and livers from WT and KO mice (*n* = 6–7 per group). (**B**) mRNA expression of *ZFP90* in MT-2 and SKW-3 cells (*n* = 3 per group). (**C**) mRNA expression of *ZFP90, TNFα, IL2TA, and IL2RB* in MT-2 cells transfected with control or *ZFP90*-siRNA (*n* = 3 per group). (**D**) Representative immunoblot analysis of p65 and Lamin B1 protein levels in the livers from WT and KO mice (*n* = 4 per group). Data are expressed as means ± SEM. Statistical significance was determined by Student’s two-tailed *t*-test; * *p* < 0.05, ** *p* < 0.01, and *** *p* < 0.001.

Data from the Human Protein Atlas indicate high expression of both ZFP90 and TRIM28 in liver immune cells, particularly T cells [28]. To further elucidate the hepatic immunomodulatory function of ZFP90, we measured the mRNA expression levels of T-cell markers (*Cd4* and *Cd8a*), as well as *Tlr7*, *Trim28*, and *Tnfa*. ZFP90 deficiency significantly altered the intrahepatic immune landscape, characterized by reduced *Cd4* expression alongside increased *Tlr7* and *Tnfa* expression levels (Figure 5A). Collectively, these findings suggest that ZFP90 is essential for maintaining hepatic T-cell homeostasis, and its absence shifts the immune microenvironment toward a pro-inflammatory state during metabolic stress. To evaluate whether this local shift reflects a broader systemic alteration linked to the ZFP90-TRIM28 axis, we analyzed the peripheral blood of the whole-body ZFP90-KO mice. Notably, we observed a significant reduction in the circulating T-cell population in the KO mice compared to WT controls (Figure S6). Given that TRIM28 is essential for T-cell proliferation and survival [29], this systemic decrease provides functional *in vivo* evidence that the loss of ZFP90 phenocopies aspects of TRIM28 impairment, thereby functionally linking the ZFP90-TRIM28 interaction to T-cell dynamics.

To investigate the specific role of ZFP90 in T cells, we examined the mRNA expression profiles of T-cell-related genes in MT2, a human regulatory T (Treg)-like cell line [30], and SKW3, a T-cell leukemia cell line. These cell lines exhibited distinct phenotypic characteristics; MT2 cells showed high expression of *IL2RA* and *IL2RB*, whereas SKW3 cells displayed elevated levels of *TNFA* and *IL2* (Figure S7). Notably, ZFP90 expression was more than two-fold higher in MT2 cells than in SKW3 cells (Figure 5B). Since TRIM28 plays a crucial role in Treg cell function [29], we performed ZFP90 knockdown in MT2 cells to evaluate its impact. Interestingly, the knockdown of ZFP90 markedly increased the mRNA expression of *TNFα* (Figure 5C). Consistent with these findings, we observed significantly elevated nuclear p65 protein levels and reduced cytosolic IκBα protein levels in the livers of KO mice compared to WT controls (Figure 5D). These results suggest that ZFP90 may function as a transcriptional repressor of pro-inflammatory cytokines, likely by cooperating with TRIM28 to maintain the immunosuppressive identity of Treg cells.

### 3.6. Hepatic ZFP90 Expression Correlates with MASLD Severity in Humans

**Figure 6:**
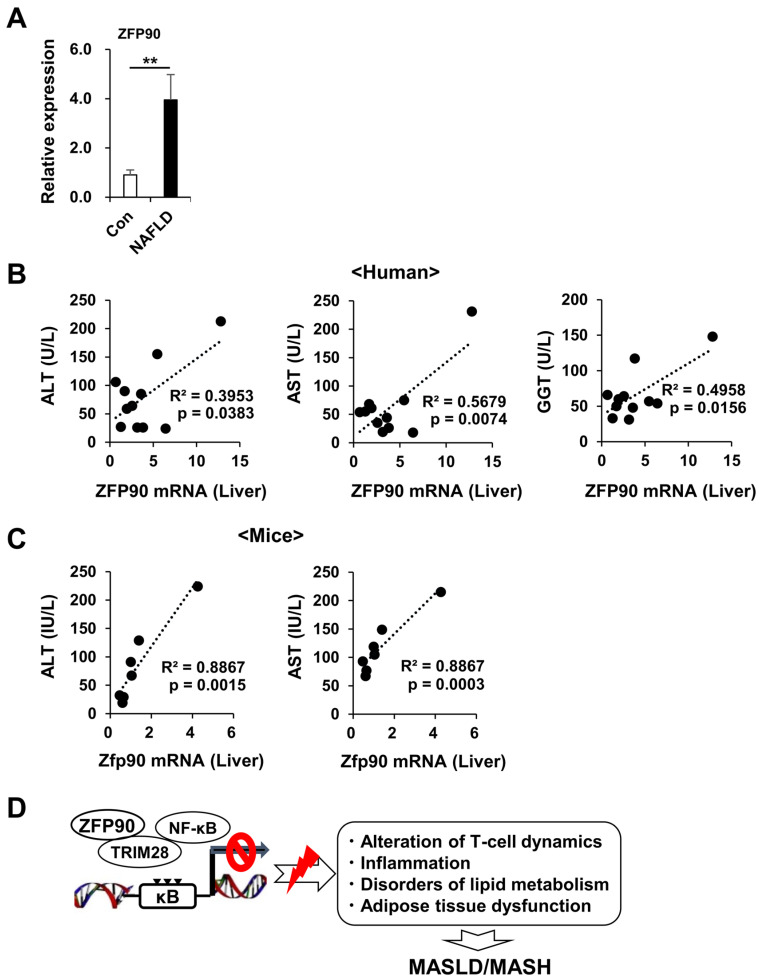
Clinical correlation of ZFP90 with MASLD severity. (**A**) mRNA expression of ZFP90 in the livers of healthy controls and patients with MASLD (*n* = 11–12 per group). (**B**,**C**) Correlation between *Zfp90* mRNA levels and serum ALT, AST, and GGT levels in (**B**) human MASLD patients and (**C**) HFD-fed mice (*n* = 11–12 per group). (**D**) Schematic summary: ZFP90 functions as a pivotal regulator of hepatic immunometabolic homeostasis. ZFP90 deficiency exacerbates MASLD/MASH through a multi-hit process involving lipid metabolism disorders, adipose tissue dysfunction, hyper-activation of NF-κB–dependent inflammatory signaling, and altered T-cell dynamics. Data are expressed as means ± SEM. Statistical significance was determined by Student’s two-tailed *t*-test; ** *p* < 0.01.

Finally, we assessed the clinical relevance of ZFP90 in humans. Table 1 summarizes the physical, anthropometric, and biochemical evaluations, including biomarkers of MASLD, for both patients and controls across all study phases. We first examined the hepatic mRNA expression of *ZFP90* and found that it was significantly elevated in patients with MASLD compared to healthy controls (Figure 6A). Furthermore, we explored the association between ZFP90 expression and individual biomarkers in the MASLD cohort. Consistent with our murine data, *ZFP90* expression showed a strong positive correlation with serum AST and ALT concentrations (Figure 6B,C). Additionally, *ZFP90* levels were positively correlated with γ-GTP levels (Figure 6B). This clinical upregulation of *ZFP90* expression likely reflects a compensatory, albeit insufficient, physiological response to chronic adipo-hepatic injury and severe metabolic stress.

## 4. Discussion

The present study identified ZFP90 as a novel transcriptional regulator that governs the intricate balance between hepatic lipid metabolism and inflammatory signaling under conditions of nutrient overload. Through ZFP90-deficient murine models and clinical validation, we demonstrated that the loss of this zinc finger protein significantly accelerates the transition from simple steatosis to an aggressive MASH-like phenotype. Our findings provide compelling evidence that ZFP90, via its physical and functional interaction with TRIM28, serves as a crucial molecular brake in the NF-kB signaling pathway, thereby protecting the liver from obesity-induced metabolic lipotoxicity and chronic inflammation.

Crucially, our study highlights the profound impact of ZFP90 deficiency on systemic metabolic homeostasis, primarily driven by adipose tissue dysfunction. Under chronic dietary stress, the observed imbalance between lipogenic and lipolytic processes, coupled with impaired insulin signaling (reduced *p*-Akt), suggests that ZFP90 is essential for maintaining healthy adipose expansion. Dysfunctional adipose tissue directly contributes to hepatic steatosis via the adipo-hepatic axis, where increased lipolysis and pro-inflammatory adipokine secretion drive systemic lipotoxicity and hepatic insulin resistance. These systemic alterations act in concert with intrahepatic mechanisms to accelerate MASLD progression (Figure 6D).

Consequently, the influx of adipose-derived lipids and inflammatory signals exacerbates hepatic lipotoxicity. We found that ZFP90 deficiency promotes hepatic steatosis independently of caloric intake (Figure S1), suggesting a direct regulatory role in hepatic lipid handling in response to systemic overflow. Transcriptomic profiling revealed that ZFP90 deficiency leads to the robust induction of genes involved in fatty acid uptake (*Cd36*, *Mogat1*) and pro-inflammatory cascades (*Ccl2*, *Tnfa*). This dual dysregulation indicates that ZFP90 is not merely a metabolic regulator but a master scaffold that integrates extracellular nutrient sensing with local immune responses. Specifically, the induction of *Ccr2* and *Cd68* in *Zfp90*-KO livers underscores the increased recruitment of monocyte-derived macrophages, which are fundamental drivers of the persistent inflammation characterizing MASLD progression.

The mechanistic nexus between ZFP90 and TRIM28 identified here adds a new layer of complexity to the known functions of Krüppel-associated box domain (KRAB)-ZFPs in the liver. While KRAB-ZFPs typically recruit TRIM28 for epigenetic silencing, their roles in hepatic metabolic diseases remain understudied. Our data suggest that the ZFP90-TRIM28 complex acts as a repressive unit that restricts the nuclear translocation and transcriptional activity of p65. This aligns with prior reports indicating that TRIM28 can sequester the NF-κB (p65) subunit to dampen inflammatory output [27]. Dysregulated NF-κB signaling is a well-established driver of MASLD, mediating hepatocyte injury and macrophage activation [31,32]. The loss of ZFP90 effectively “relieves the brake” in this system, priming the liver for a hyper-inflammatory response to nutrient-induced metabolic stress. This inflammatory priming is further corroborated by a significant shift in intrahepatic T-cell dynamics, specifically the reduced *Cd4* and increased *Tnfa* [14,33,34,35,36,37]. We observed that ZFP90 deficiency drives this shift, likely through NF-κB-dependent chemokine signaling, which amplifies local inflammation and hepatocellular injury. Furthermore, it is well-established that TRIM28 is a critical factor for T-cell proliferation, survival, and regulatory function [30]. In our study, the profound reduction in circulating T-cells observed in the peripheral blood of ZFP90-KO mice (Figure S6) directly mirrors the established consequences of TRIM28 impairment. We interpret this systemic T-cell reduction as a downstream consequence of compromised TRIM28 function resulting from the absence of its binding partner, ZFP90. This *in vivo* phenocopy strongly substantiates the functional interdependency of the ZFP90-TRIM28 complex in maintaining both systemic and local T-cell homeostasis.

Furthermore, our clinical data provide a critical translational perspective. At first glance, the significant upregulation of hepatic ZFP90 in patients with MASLD might appear contradictory to the severe disease phenotype observed in our ZFP90-KO mice. However, this observation is consistent with our in vivo findings: we observed a significant increase in ZFP90 expression in high-fat diet (HFD)-fed mice, suggesting that this upregulation is an evolutionarily conserved response to metabolic overload. Rather than driving the pathogenesis, we interpret this elevated expression as a compensatory, adaptive defense mechanism against ongoing metabolic injury. Similar to the upregulation of other protective factors during tissue stress, the liver likely attempts to upregulate ZFP90 to apply the “transcriptional brake” on NF-κB and mitigate hyper-inflammation under chronic systemic metabolic stress. The strong positive correlation between ZFP90 mRNA and serum AST/ALT levels suggests that as lipotoxicity and tissue damage progress, this endogenous regulatory capacity is eventually overwhelmed, rendering the compensatory upregulation insufficient to halt MASH progression. Despite these findings, this study is subject to several limitations that deserve further consideration. First, the tissue-specific contributions of ZFP90 (such as in adipocytes versus hepatocytes) should be rigorously investigated using conditional knockout models. Second, while our *in vitro* data utilizing the Treg-like MT-2 cell line provides preliminary mechanistic insights, its HTLV-1 transformed nature remains a limitation. Therefore, these ZFP90-dependent transcriptional changes must be validated in primary murine or human peripheral T cells, and the precise downstream effectors of the ZFP90-TRIM28 complex require further characterization. Third, our analysis of the intrahepatic immune landscape currently relies primarily on transcriptomic profiles rather than direct cellular quantification. As bulk mRNA analysis cannot delineate changes in specific immune cell numbers, subset composition, or activation states, and markers such as *Tlr7* are not strictly T-cell specific, our current data reflect a generalized inflammatory transcriptomic shift rather than definitive alterations in T-cell dynamics. Future studies utilizing flow cytometry or single-cell RNA sequencing are required to rigorously map the specific immune cell subpopulations infiltrating the ZFP90-deficient liver under dietary stress. Fourth, this initial study exclusively utilized male mice to avoid the confounding metabolic variations introduced by the estrous cycle during the prolonged 20-week high-fat diet regimen. Given the well-established sex differences in MASLD pathogenesis, future studies evaluating female ZFP90-KO mice are necessary to determine if the protective role of ZFP90 is sexually dimorphic. Finally, our mechanistic mapping of the ZFP90-TRIM28 interaction was primarily conducted using an in vitro overexpression system (COS-7). While demonstrating this interaction endogenously within liver tissue would be ideal, it was technically precluded by the limited immunoprecipitation efficiency of currently available ZFP90 antibodies and the high non-specific background inherent to lipid-rich hepatic lysates. Nevertheless, the pronounced hyper-activation of NF-κB signaling observed in our primary ZFP90-deficient NPCs and in vivo models strongly supports the physiological relevance of this repressive complex. Future studies should focus on the pharmacological modulation of the ZFP90 pathway to evaluate its clinical utility in treating obesity-associated metabolic complications.

## 5. Conclusions

This study establishes ZFP90 as a critical gatekeeper of systemic and hepatic immunometabolic integrity under dietary stress. By elucidating the adipo-hepatic interplay and the role of ZFP90 in inflammatory signaling, we uncovered a potential therapeutic target that could be exploited to arrest MASLD progression. Strategies aimed at stabilizing ZFP90 activity or enhancing its interaction with co-repressors, such as TRIM28, may offer a novel avenue for mitigating systemic lipotoxicity and preventing the transition to advanced MASH.

## Figures and Tables

**Table 1:** Clinical and biochemical characteristics of the participants.

Variable (Mean ± SD)	Non-MASLD Control Subjects	MASLD Subjects	*p* Value
Number of subjects	12	11	
Female/male, %	50/50	64/36	
Age, years	50.89 ± 9.36	56.73 ± 10.57	NS
BMI, kg/m^2^	20.57 ± 0.82	29.34 ± 5.30	<0.00002
Fasting plasma glucose, mg/dL	93.25 ± 3.96	115.50 ± 28.49	<0.02
SABP, mm Hg	108.42 ± 13.27	123.82 ± 15.91	<0.02
DABP, mm Hg	73.67 ± 15.42	71.73 ± 8.06	NS
*C*-reactive protein, mg/dL	0.03 ± 0.03	0.16 ± 0.08	<0.00004
HDL-cholesterol, mg/dL	80.33 ± 28.07	51.55 ± 18.01	<0.009
LDL-cholesterol, mg/dL	134.42 ± 25.2	109.27 ± 31.98	<0.05
Triglyceride, mg/dL	76.50 ± 23.00	170.09 ± 69.40	<0.0003
ALT, U/L	15.33 ± 7.89	79.55 ± 60.49	<0.002
AST, U/L	17.25 ± 3.02	62.36 ± 59.17	<0.02
GGT, U/L	15.75 ± 6.55	66.18 ± 35.33	<0.00009

Results are expressed as mean ± SD. ALT and AST, serum alanine and aspartate aminotransferase; BMI, body mass index; DABP, diastolic arterial blood pressure; GGT, γ-glutamyl-transferase; HDL, high-density lipoprotein; LDL, low-density lipoprotein; NS, non-significant; SABP, systolic arterial blood pressure.

## Data Availability

Transcriptomic (RNA-seq) data from this study have been deposited in the NCBI Gene Expression Omnibus (GEO) under accession number GSE324098.

## Supplementary Materials

The following supporting information can be downloaded at: https://www.mdpi.com/article/10.3390/nu18142332/s1, Figure S1: ZFP90 deficiency modulates the dietary effect on food intake; Figure S2: ZFP90 deficiency does not affect adipocyte size in white adipose tissue; Figure S3: ZFP90 deficiency alters hepatic gene expression profiles related to immunity and lipid metabolism; Figure S4: ZFP90-deficiency does not affect glucose tolerance; Figure S5: mRNA expression levels in liver. mRNA expression of *Cd36*, *TNFα*, and *F4/80* in liver tissue from CRF-1 and HFD-fed mice; Figure S6: Systemic hematological analysis of ZFP90-deficient mice; Figure S7: mRNA expression levels in MT-2 and SKW-3 cells; Table S1: Gene name, symbols, and primer sequences (Mus musculus) for quantitative real-time PCR; Table S2: Gene name, symbols, and primer sequences (Homo sapiens) for quantitative real-time PCR.
