# Supplementary material for: ZFP90 Serves as a Transcriptional Brake on NF-κB Signaling to Attenuate Diet-Induced MASLD Progression
Source: Nutrients. 2026 Jul 16;18(14):2332. doi: 10.3390/nu18142332 (PMC13414558; doi:10.3390/nu18142332)

Supplementary information

Materials and methods

Glucose Tolerance Test (GTT)

Glucose tolerance tests were performed to assess glucose homeostasis in mice. Mice were fasted for 16 hours with free access to water. Baseline blood glucose levels were measured from tail vein blood using a Accu-Chek Aviva Nano Meter (Roche Diagnostics, Tokyo, Japan). Following baseline measurement, mice were injected intraperitoneally (i.p.) with a glucose solution (1 g/kg of body weight). Blood glucose levels were subsequently measured at 15, 30, 60, and 120 minutes post-injection.

Blood Collection and Analysis

To investigate the systemic inflammatory response in vivo, ZFP90 WT and KO mice were injected intraperitoneally with lipopolysaccharide (LPS, 0.5 mg/kg body weight). Two hours post-injection, mice were deeply anesthetized with isoflurane. Blood samples were collected via cardiac puncture using a 25-gauge needle into tubes. Hematological parameters were measured immediately using the Thinka blood analyzer (ARKRAY, Inc., Kyoto, Japan) according to the manufacturer's instructions. All procedures were conducted to minimize distress, and the total blood volume collected was strictly maintained within institutional animal welfare limits.

Figure Legends of supplementary data

Supplementary Figure S1. ZFP90 deficiency modulates the dietary effect on food intake. Food intake was measured in mice fed either a standard diet (CRF-1) or a high-fat diet (HFD) (n = 3 per group). Data are presented as mean ± SEM. Statistical significance was determined by three-way ANOVA with Bonferroni's multi-ple-comparisons test or Student's two-tailed t-test.

Supplementary Figure S2. ZFP90 deficiency does not affect adipocyte size in white adipose tissue (n = 4 per group). Adipocyte sizes were compared in epididymal WAT (eWAT) and inguinal WAT (iWAT) white adipose tissue (n = 4 per group). Data are presented as mean ± SEM. Statistical significance was determined by Student's two-tailed t-test; $^{*}p < 0.05$, $^{**}p < 0.01$, and $^{***}p < 0.001$.

Supplementary Figure S3. ZFP90-deficiency alters hepatic gene expression profiles related to immunity and lipid metabolism. Transcriptomic analysis revealed changes in pathways involving inflammatory responses, cytokine-cytokine receptor interactions, NF-κB, and chemokine signaling. (A) Volcano plots highlighting differentially expressed genes (DEGs). (B) KEGG pathway enrichment analysis of the identified DEGs. (A, B) Analyses were performed using a threshold of FC < 2.0 and $P_{adj} < 0.05$ (via iDEP2.4.4 and DAVID bioinformatics tools) ($n = 3$ per group).

Supplementary Figure S4. ZFP90-deficiency does not affect glucose tolerance. GTT from WT and KO mice (n = 6-7 per group). Data are presented as mean ± SEM. Statistical significance was determined by three-way ANOVA with Bonferroni's multi-ple-comparisons test or Student's two-tailed t-test.

Supplementary Figure S5. mRNA expression levels in liver. mRNA expression of *Cd36, TNFα,* and *F4/80* in liver tissue from CRF-1 and HFD-fed mice (n = 6-7 per group). Data are presented as mean ± SEM. Statistical significance was determined by Student's two-tailed t-test; $^{**}p < 0.01$.

Supplementary Figure S6. Systemic hematological analysis of ZFP90-deficient mice. Peripheral blood was collected via cardiac puncture 2 h after LPS injection (0.5 mg/kg, i.p.). Hematological parameters, including white blood cells (WBC), lymphocytes (Lym), monocytes (Mon), and granulocytes (Gra), were measured using the Thinka blood analyzer (n = 6 per group). Data are presented as mean ± SEM. Statistical significance was determined by Student's two-tailed t-test; $^{*}p < 0.05$.

Supplementary Figure S7. mRNA expression levels in MT-2 and SKW-3 cells. mRNA expression of genes related to T cells (*TNFα, IL2, IL2RA, IL2RB, FOXP3*). Data are presented as mean ± SEM. Statistical significance was determined by Student's two-tailed t-test; $^{**}p < 0.01$, and $^{***}p < 0.001$.

Figure S1.

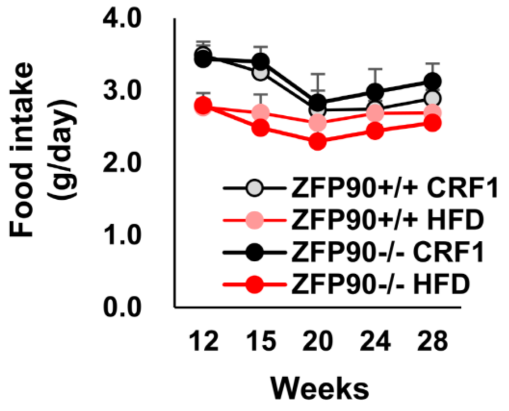

Figure S2.

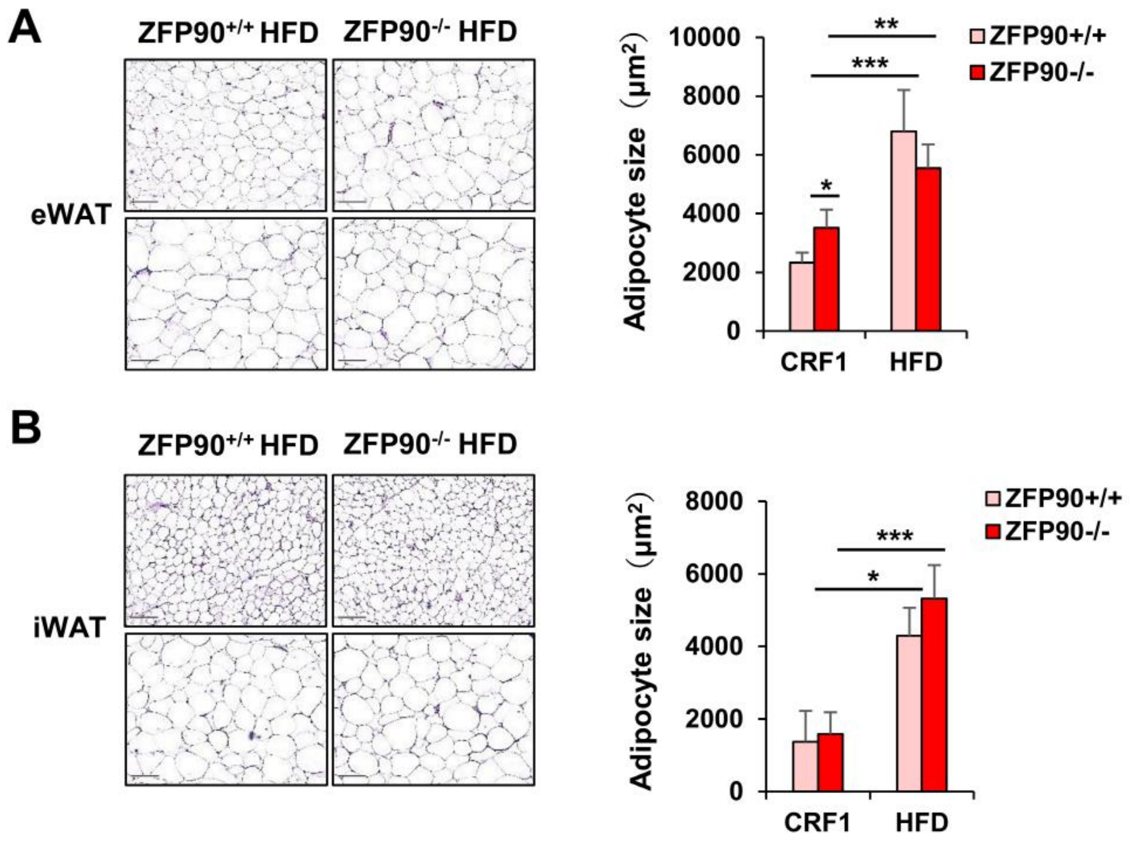

Figure S3.

A

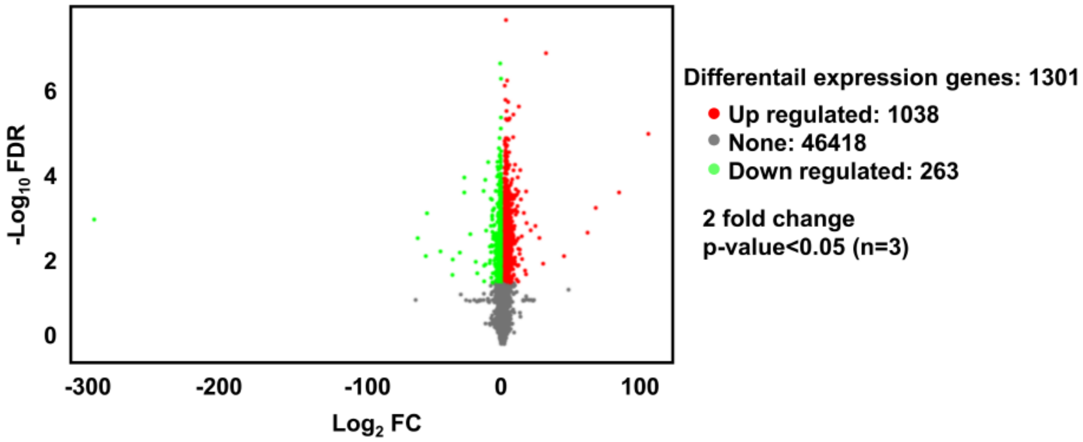

B

KEGG_Pathway

Term	Count	P-value
Cytokine-Cytokine receptor interaction	40	4.7E-9
Focal adhesion	30	8.4E-8
Cell adhesion molecules	25	4.7E-6
NF-kB signaling pathway	18	1.1E-5
Lipid and atherosclerosis	26	3.5E-5
Chemokine signaling pathway	24	4.6E-5

Figure S4.

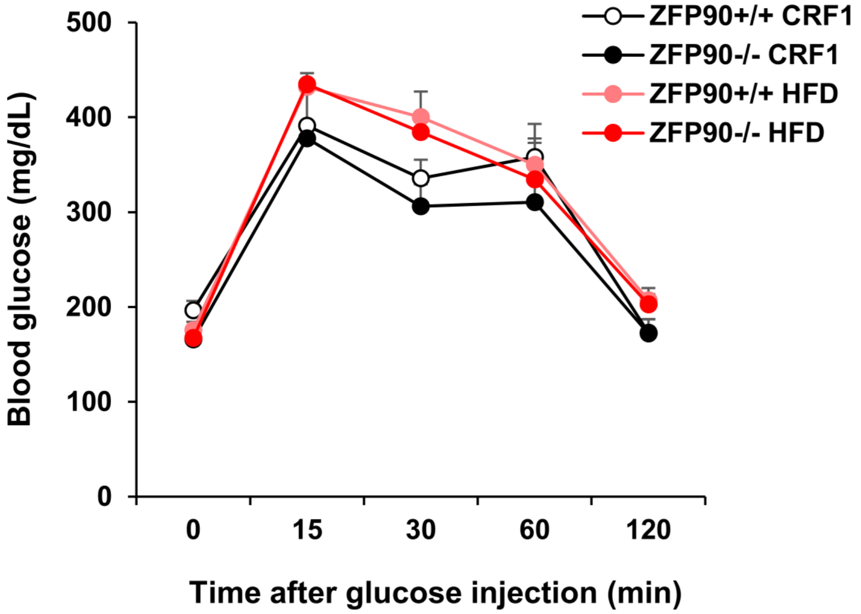

Figure S5.

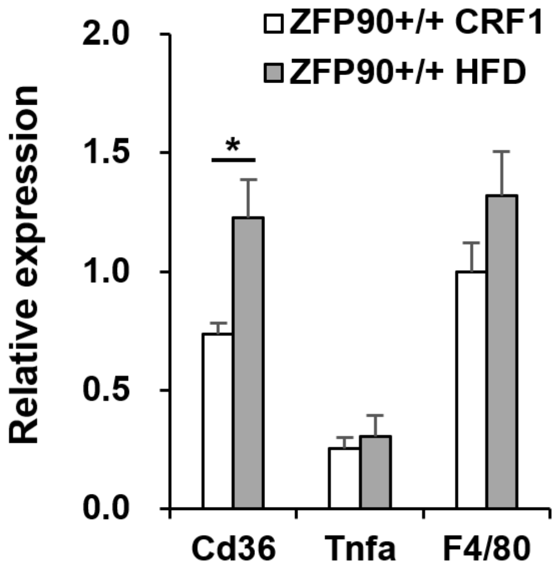

Figure S6.

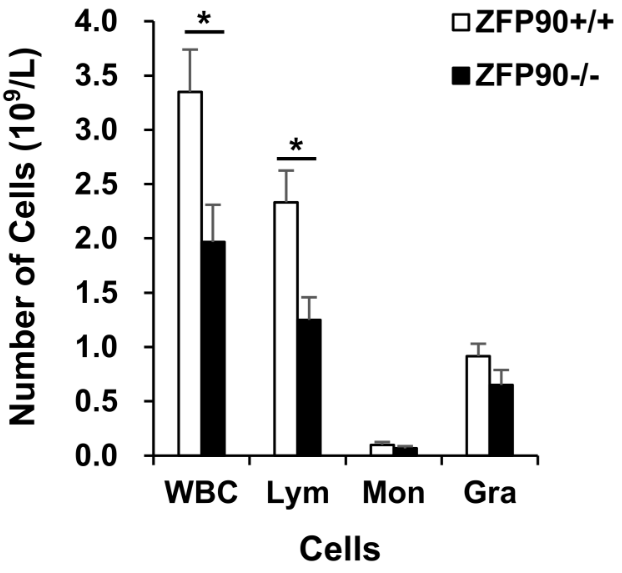

Figure S7.

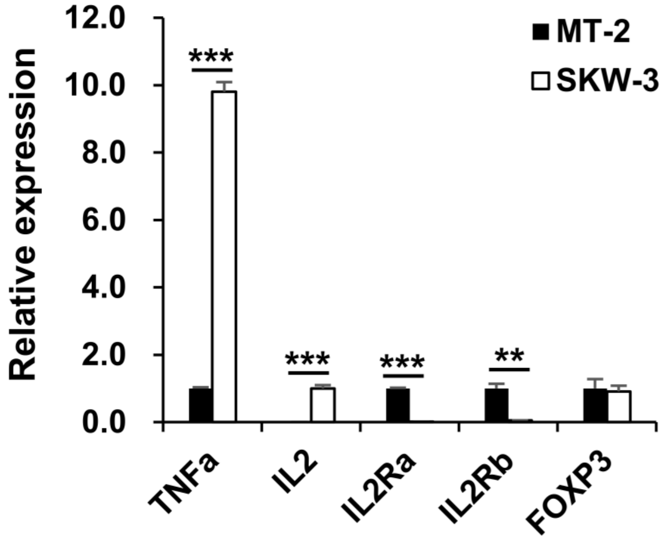

Supplement: Supplementary file 1 [file nutrients-18-02332-s001.zip › Supplementary files.pdf]
