# Supplementary material for: ZFP90 Serves as a Transcriptional Brake on NF-κB Signaling to Attenuate Diet-Induced MASLD Progression
Source: Nutrients. 2026 Jul 16;18(14):2332. doi: 10.3390/nu18142332 (PMC13414558; doi:10.3390/nu18142332)
Supplement: Supplementary file 1 [file nutrients-18-02332-s001.zip › Table S1.pdf]

Table S1. Gene name, symbols, and primer sequences (Mus musculus).

Gene Name	Gene Symbol	Forward Primer	Reverse Primer
18s ribosomal RNA	*18S*	TTCTGGCCAACGGTCTAGACAAC	CCAGTGGTCTTGGTGTGCTGA
Ribosomal protein lateral stalk subunit P0	*Rplp0*	TCATCCAGCAGGTGTTTGACA	GGCACCGAGGCAACAGTT
Acetyl-Coenzyme A	*Acc1*	TGGCAGACCACTATGTTCCA	GTTCTGGGAGTTTCGGGTTC
Adiponectin	*Adipoq*	TCTGTCTGTACGATTGTCAGTGGA	GGCATGACTGGGCAGGATTA
Carbohydrate responsive element binding protein alpha	*ChREBP-α*	CGACACTCACCCACCTCTTC	TGTTCAGCCGGATCTTGTC
Chemokine ligand 2	*Ccl2*	TTAAAAACCTGGATCGGAACCAA	GCATTAGCTTCAGATTTACGGGT
Chemokine ligand 3	*Ccl3*	TTCTCTGTACCATGACACTCTGC	CGTGGAATCTTCCGGCTGTAG
C-C motif chemokine receptor 2	*Ccr2*	TGTGGGACAGAGGAAGTGG	GGAGGCAGAAAATAGCAGCA
CD36 molecule	*Cd36*	TCCTCTGACATTTGCAGGTCTATC	AAAGGCATTGGCTGGAAGAA
CD4 antigen	*Cd4*	GTTCAGGACACCGACTTCTGGA	GAAGGAGAACTCCGCTGACTCT
CD68 antigen	*Cd68*	GCCCGAGTACAGTCTACCTGG	AGAGATGAATTCTGCGCCAT
CD8 subunit alpha	*Cd8a*	ACTACCAAGCCAGTGCTGCGAA	ATCACAGGCGAAGTCCAATCCG
collagen, type I, alpha	*Col1a2*	CCGTGCTTCTCAGAACATCA	CTTGCCCCATTCATTTGTCT
Fatty acid synthase	*Fasn*	AAGGCTGGGCTCTATGGATT	GGAGTGAGGCTGGGTTGATA
Fatty acid transporter protein 1	*Fatp1*	CGCTTTCTGCGTATCGTCTG	GATGCACGGGATCGTGTC T
Fatty acid transporter protein 4	*Fatp4*	ACTGTTCTCCAAGCTAGTGCT	GATGAAGACCCGGATGAAACG
Lysosomal acid lipase A	*Lipa*	TGACTGACTAGCAAGCGTCCACA A	ATTACCTCCACACAAGGGCCAGA A
Lipoprotein lipase	*Lpl*		
Monoacylglycerol O-acyltransferase 1	*Mogat1*	CCAGCACTACTTTGGCATAATGC	CCTCTAGCTATGTCTGATGCAGC
Peroxisome proliferator-activated receptor gamma 2	*Pparγ2*	TGGGTGAAACTCTGGGAGAT	GCTGGAGAAATCAACTGTGG
Toll-like receptor 7	*Tlr7*	TTCCTTCCGTAGGCTGAACC	GTAAGCTGGATGGCAGATCC
Tumor necrosis factor alpha	*Tnfα*	CCCTCACACTCAGATCATCTTCT	GCTACGACGTGGGCTACAG
Tripartite motif-containing 28	*Trim28*	TGGACCAAGAGTGCTGAAGCCT	CTACTGCCAGAACCTTGCTTGC
