# Supplementary material for: ZFP90 Serves as a Transcriptional Brake on NF-κB Signaling to Attenuate Diet-Induced MASLD Progression
Source: Nutrients. 2026 Jul 16;18(14):2332. doi: 10.3390/nu18142332 (PMC13414558; doi:10.3390/nu18142332)
Supplement: Supplementary file 1 [file nutrients-18-02332-s001.zip › Table S2.pdf]

Table S2. Gene name, symbols, and primer sequences (Homo sapiens).

Gene Name	Gene Symbol	Forward Primer	Reverse Primer
Glyceraldehyde-3-phosphate dehydrogenase	*GAPDH*	TGAACGGGAAGCTCACTGG	TGAACGGGAAGCTCACTGG
Interleukin 2 receptor subunit alpha	*IL2RA*	ATCAGTGCGTCCAGGGATAC	GTGACGAGGCAGGAAGTCTC
Interleukin 2 receptor subunit beta	*IL2RB*	GCTGATCAACTGCAGGAACA	TGTCCCTCTCCAGCACTTCT
Tumor necrosis factor alpha	*TNFα*	CTCTTCTGCCTGCTGCACTTTG	ATGGGCTACAGGCTTGTCACTC
